# Synthesis of bone biocompatible implants using human adipose-derived mesenchymal stem cells (hADMSCs) and PCL/laminin scaffold substrate

**DOI:** 10.22038/IJBMS.2023.71307.15491

**Published:** 2024

**Authors:** Donya Zeydari, Ehsan Karimi, Ehsan Saburi

**Affiliations:** 1 Department of Biology, Mashhad Branch, Islamic Azad University, Mashhad, Iran; 2 Medical Genetics Research Center, School of Medicine, Mashhad University of Medical Sciences, Mashhad, Iran; 3 Medical Genetics and Molecular Medicine Department, School of Medicine, Mashhad University of Medical Sciences, Mashhad, Iran

**Keywords:** Laminin, Mesenchymal stem cells, Osteogenesis, Polycaprolactone, Tissue engineering

## Abstract

**Objective(s):**

Bone tissue engineering is considered a new method in the treatment of bone defects and can be an effective alternative to surgery and bone grafting. The use of adipose tissue mesenchymal stem cells (ADMSCs) on synthetic polymer scaffolds is one of the new approaches in bone tissue engineering. In this study, we aimed to investigate the effect of laminin coating on biocompatibility and osteogenic differentiation of ADMSCs seeded on polycaprolactone (PCL) scaffolds.

**Materials and Methods:**

The morphology of the electrospun scaffold was evaluated using a scanning electron microscope (SEM). Cell proliferation and cytotoxicity were determined by MTT assay. The adipogenic and osteogenic differentiation potential of the cells was evaluated. The osteogenic differentiation of ADMSCs cultured on the PCL scaffold coated with laminin was assessed by evaluating the level of alkaline phosphatase (ALP) activity, intracellular calcium content, and expression of bone-specific genes.

**Results:**

The results showed that the ADMSCs cultured on PCL/laminin showed enhanced osteogenic differentiation compared to those cultured on non-coated PCL or control medium (*P*<0.05).

**Conclusion:**

It seems that laminin enhances the physicochemical properties and biocompatibility of PCL nanofiber scaffolds; and by modifying the surface of the scaffold, improves the differentiation of ADMSCs into osteogenic cells.

## Introduction

Tissue engineering has been developed to replace or repair damaged organs (1). Bone tissue engineering (BTE) is a branch of a tissue engineering approach that allocates new procedures to treat bone defects (2). Tissue engineering relies on three main components: stem cells, scaffolds, and growth factors, which provide cell/tissue source, extracellular matrix, and messenger molecules, respectively (3). 

Stem cells are a population of immature and tissue precursor cells with self-renewal potency that can differentiate into different types of adult cells in appropriate conditions (4). Stem cells can be isolated from various sources such as skin, bone marrow, fat, umbilical cord, hair follicles, tooth muscles, tooth pulp, and periodontal fibers (5). 

Mesenchymal stem cells are multipotent stromal stem cells with a high proliferation rate suitable for tissue engineering (3). Bone marrow-derived mesenchymal stem cells (BMMSCs) can be used to treat osteoporosis. However, the isolation and use of these cells have limitations, including the need for frequent general or spinal anesthesia, the cost and imposing pain during the cell isolation process, and the low harvesting rate (approximately one cell per 10^5^ stromal cells) (1, 3). On the other hand, adipose-derived mesenchymal stem cells (ADMSCs) are molecularly similar to bone marrow mesenchymal stem cells and are also an available and ideal source of autologous stem cells (6).

The extracellular matrix provides the cell with various survival, growth, migration, and differentiation functions and maintains normal tissue homeostasis (7). Synthetic and natural polymers are used in tissue engineering as the nanofibrous scaffold, mimicking the ECM structure and function to restore, maintain, or improve human tissue function (8). Moreover, these nanofibers have a high surface-to-volume ratio and excellent tensile strength (9). Polymeric nanofibers increase the uptake of specific proteins such as fibronectin and vibronectin, promoting cell attachment and expansion (2, 10). Due to the piezoelectric property of bone tissue, recruited scaffolds must share high mechanical strength and piezoelectric properties (3) together with high biocompatibility, biodegradability, and suitable pore size (11).

Poly-caprolactone (PCL), a synthetic polymer with potential applications in tissue engineering and regenerative medicine, was used as a cell scaffold to mimic the function of ECM. However, several studies are being conducted to improve the biocompatibility, biodegradability, and tissue compatibility of this polymer. Among the most important compounds used for this purpose are natural polymers such as fibronectin, laminin, fibrin, etc (12, 13). Appropriate biocompatibility and biodegradability, good pore size, in-range mechanical flexibility, high surface-to-volume ratio, and non-toxicity can be provided by PCL (11). On the other hand, one of the components of the natural ECM is ​​laminin, which binds to the membrane receptor, adhering cells to the substrate, and increases cell adhesion, growth, and differentiation (14). Structurally, laminin has three proteins, alpha, beta, and gamma, which form the structure of laminin (15, 16). Laminin is used to improve the hydrophilicity and bioactive properties of the polymer to optimize the binding, proliferation, and differentiation of mesenchymal stem cells on the scaffold (4, 17). Therefore, we aimed to fabricate laminin-coated PCL scaffolds to enhance the osteogenic differentiation of ADMSCs.

## Materials and Methods

***Nanofibrous scaffold preparation***

The electrospinning technique has been used to fabricate nanofibrous scaffolds. First, 1.2 g of PCL polymer (Sigma-Aldrich, St. Louis, MO, USA, MW=80,000g/mol) was dissolved in chloroform and dimethylformamide (DMF) solution (Merck, Berlin, Germany). Next, the polymeric solution was electrospuned with a syringe at a distance of 20 cm from the collector, a flow rate of 0.5 ml per hour, and a voltage of 20 kV.

***Mechanical properties***

The mechanical properties of the fabricated scaffold were evaluated through the tensile test using an Instron device (Model STM -20; SANTAM, Tehran, Iran) at a speed of 50 mm/min. The data were obtained and plotted as a stress pressure curve.

***Morphological study***

Scanning electron microscopy (SEM, S - 4500; Hitachi, Tokyo, Japan) was used to evaluate the morphology of the scaffold and cultured cells. Cell-cultured nanofiber samples were fixed with 2.5% glutaraldehyde and then dried using an ethanol serial dilution (from 50° to 100°). The scaffolds were transferred to aluminum bases and covered with a layer of gold. Then, the free and seeded scaffolds were imaged by a specialized operator. 

***Stem cell isolation***

Adipose tissue-derived mesenchymal stem cells (AD-MSCs) were used in the present study. AD-MSCs were isolated and purified according to previous studies (1). After isolation from adipose tissue, the cells were transferred to a flask containing a high-glucose DMEM culture medium supplemented with 15% bovine fetal serum (FBS) for cell proliferation. Then trypsin 0.25% was used for cell passage. The AD-MSCs were cultured in passage 3 in a DMEM low glucose base medium with 10% FBS and 1% antibiotic (to prevent possible contamination) and incubated in an incubator at 37 °C, 5% CO_2_, and 96% humidity. ADMSCs were seeded on the nanofibrous scaffold after reaching 70%–80% confluency for differentiation into bone cells.

***Cell culture***

The PCL scaffolds were sterilized by exposure to UV radiation and 70% ethanol before cell seeding. Then, the sterilized scaffolds were incubated at 37 °C for 8 hr. Then the prepared laminin (Merck, Darmstadt, Germany) was sterilized by exposing it to UV radiation for two hours in proximity to the scaffold to establish possible bonds and penetrate the scaffold pores, which would result in its slow release. AD-MSCs were seeded at a density of 10^4^/cm^2^ on the PCL scaffolds for MTT assay and SEM imaging.

***Adipogenic differentiation***

To evaluate the differentiation ability of ADMSCs, the cells were induced to differentiate into osteogenic lineage. The osteogenic medium contained 3 mM β-glycerophosphate, 50 μg/ml ascorbic acid, and 9-10 M dexamethasone. Then, 3×10^4^ cells per cm^2^ were cultured for differentiation evaluation. Two weeks after differentiation induction, the cells were stained with Alizarin Red for calcium deposition and evaluated for alkaline phosphatase activity and osteogenic gene expression.

***Survival assay***

MTT (3-(4,5-dimethylthiazol-2-yl)-2,5-diphenyltetrazolium bromide), a colorimetric method, was used to measure cell proliferation and viability. This experiment was performed to evaluate the toxicity of the PCL/laminin scaffold and its effect on the ADMSCs proliferation and viability. For this purpose, cells were cultured in a 48-well plate. Then, 5 mg/ml of MTT solution was added to the samples during 1, 3, and 5 days after induction. The plate was then incubated at 37 °C and 5% CO_2_ for 3.5 hr. The supernatant was then discarded, and dimethylsulfoxide (DMSO) solution (Merck, Darmstadt, Germany) was added to the treated cells to dissolve the formazan crystals. Finally, the optical density of the samples in the microplate reader (Bio-Tek Instruments, Inc., Winooski, VT, USA) was measured at 570 nm.

***Alizarin-red staining***

Alizarin red staining was used to detect the calcium deposits in mesenchymal cells differentiated into bone cells. In the presence of calcium, the differentiated osteocytes (bone cells) appear arched and elongated and turn reddish-brown. In short, on the tenth and twentieth days, the medium was poured out and washed with PBS. Each cell was then fixed in 4% paraformaldehyde (Merck, Germany) for 45 min at refrigerator temperature (4 °C). After washing with PBS, Alizarin Red dye (Sigma, Aldrich, St. Louis, MO, USA) was added, and the sample was incubated at room temperature for 5 to 10 min. Finally, treated cells were washed twice with PBS, and the samples were imaged with inverted light microscopy.

***Alkaline phosphatase activity***

On days 7, 14, and 21 after differentiation, the amount of produced alkaline was measured compared to the control group using an alkaline phosphatase assay kit (Pars Azmoon, Tehran, Iran). First, the cells were lysed using RIPA (Ultrasonic Disruptor, UD201, Tomy Co, LTD, Tokyo). The scaffolds were then removed, and the samples were centrifuged at 4 °C at 15,000 rpm for 15 min. Finally, the optical absorption of the supernatant contained total protein, and the microplate reader (Bio-Tek Instruments, Inc., Winooski, VT, USA) measured the optical density at 405 nm. The ALP activity was expressed as IU/mg total protein.

***Calcium content assay***

A calcium content assay kit (Pars, Azmoon, Tehran, Iran) is recruited to measure the amount of produced calcium in differentiated bone cells. HCL (N= 0.6 Merck) was added to the samples, and then the supernatant was collected. A microplate reader (Bio-Tek Instruments, Inc., Winooski, VT, USA) was used to measure the optical absorption at 570 nm.

***Gene expression***

The osteogenic differentiation of ADMSCs and the effect of PCL/laminin scaffolds on these processes were evaluated by the Real-Time PCR technique. The relative expression of four osteogenic marker genes (COL1A1, RUNX2, Osteonectin, and Osteocalcin) normalized to B_2_M (as a housekeeping gene) was measured using this technique. Cellular RNA was extracted on days 7, 14, and 21 after differentiation using an RNA extraction kit (Qiagen Valencia, CA, USA) according to the manufacturer’s protocol. The amount of RNA extracted was measured by NanoDrop (Thermo, USA). The cDNA was synthesized by reverse transcriptase (Vivantis, USA) using random hexamers. The relative expression level of osteogenic genes was measured by the Real-Time PCR technique and SBYR Green dye (TaKara Biotechnology Dalian, China) using the Rotor-Gene6000 device (Corbett, Concord, NSW, Australia). All primers were purchased from (metabion International AG) and the sequences of recruited primers are shown in Table 1. The parameters used in the PCR reaction are also listed in Table 2.

***Statistical analysis***

The experiments were performed as triple biological replications, and the results were reported as mean and standard deviation. Data obtained from the Real-Time PCR technique were also analyzed by Rest 2009 software (Technical University Munich, Germany). Finally, the data were analyzed by GraphPad Prism 6 software (Graphpad Software Inc., La Jolla, CA, USA) and using analysis of variance (ANOVA) at *P*<0.05. Bonferroni’s *post hoc* test was used for comparison between groups.

## Results

***Cell culture and differentiation***

**Figure 1:**
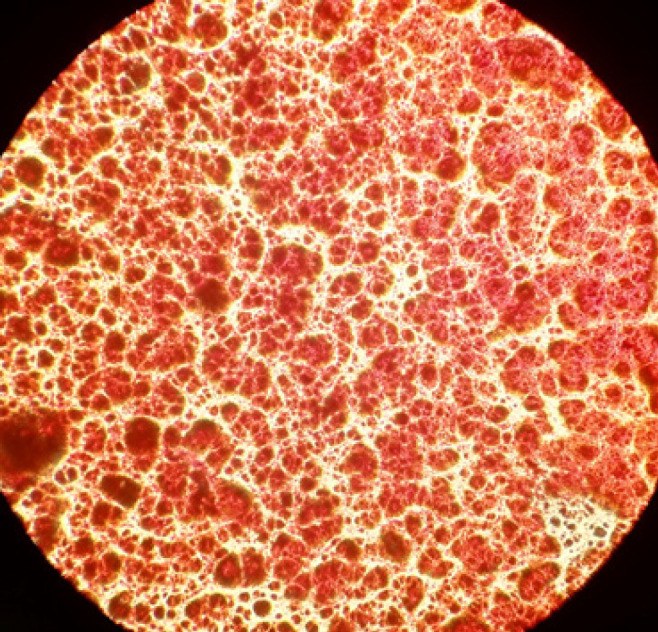
Characterization of isolated stem cells

The results showed ADMSCS at passage 4, ready to detach and seed on PCL electrospun nanofiber. Alizarin Red staining after 20 days of differentiation is also shown in Figure 1. Alizarin red staining confirmed the deposition of calcium in differentiated cells. These results indicated that ADMSCs were differentiated towards bone cells.

***SEM for scaffolds***

**Figure 2:**
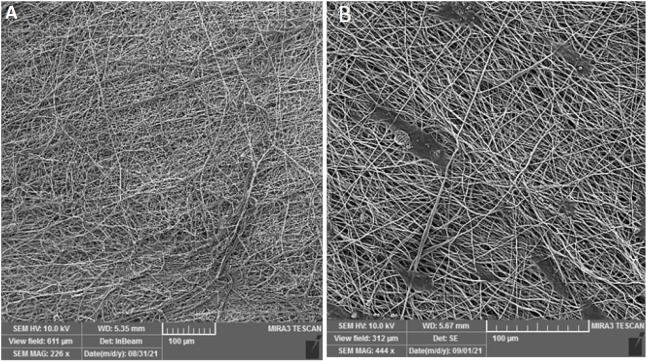
Morphology and microstructure of the electrospun scaffold before cell seeding; 226X (A) and 72 hr after cell seeding; 444X (B)

SEM images of the cell-free PCL scaffold (Figure 2A) showed that the electrospun nanofiber scaffold had a suitable pore size with a high surface-to-volume ratio. Also, the PCL/laminin scaffold was porous and had a fiber diameter of 370 nm that supported cell attachment and differentiation (Figure 2B). Then, ADMSCs were placed on a PCL/laminin. SEM images also showed the adherence of the seeded cells on the surface of the scaffold and their entrapment in the scaffold pores (Figure 2). 

***Mechanical characterization***

The tensile strength of the PCL scaffold before laminin surface modification was 36.5 ± 1.87 MPa which reached when the scaffold surface was modified with laminin. Also, the pressure at failure in the PCL scaffold was 27.5 ± 1.5% that reached following laminin modification. 

***MTT assay***

**Figure 3:**
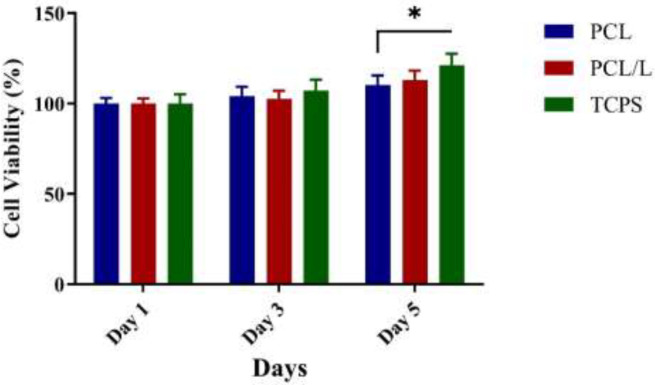
Cell viability assay results. Higher cell viability was observed in the 2D culture group after 5 days of differentiation

The MTT assay results showed that the control group showed higher cell proliferation than the PCL and PCL/laminin groups at 1, 3, and 5 days. The reason for the lower cell proliferation in the PCL/laminin scaffold was probably due to their tendency to differentiate into osteogenic cells rather than proliferate. On the other hand, the survival rate of cells on the PCL scaffold and PCL/laminin scaffold was the same in all three mentioned times and no significant difference was found between the two groups. The results also showed that the selected PCL/laminin scaffold had no toxicity to ADMSCs (Figure 3). 

***ALP activity and calcium content***

**Figure 4:**
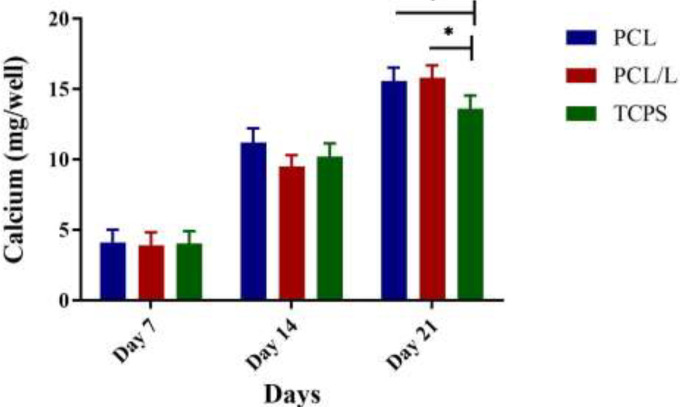
Results of the calcium content assay in different time frame and treatment conditions

**Figure 5:**
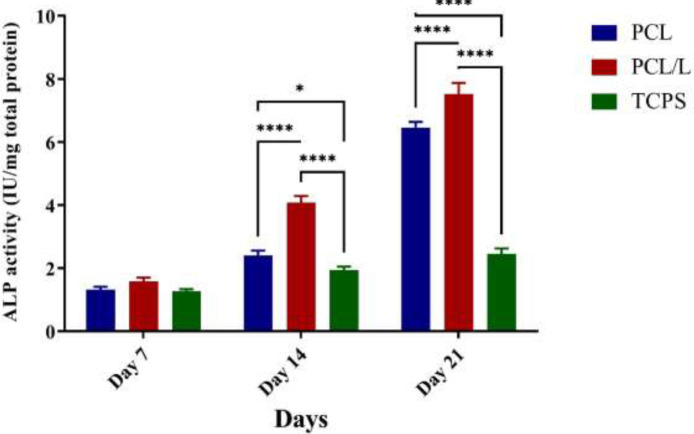
Results of ALP activity In different time frame and treatment conditions

Two important markers in osteogenic cells are ALP activity and calcium content. The level of calcium content as a major marker in osteogenic cells showed a similar trend to ALP. The level of calcium content increased from day 7 to day 21 in both PCL and PCL/laminin scaffolds compared to the control group. It was also found that the cells differentiated on the PCL/laminin scaffold had higher calcium content in comparison to cells differentiated on the PCL scaffold (Figure 4). The results of our study showed that levels of ALP activity increased significantly from day 7 to day 21 in both PCL and PCL/laminin scaffolds compared to the control group. In addition, the ALP activity in the PCL/laminin group was higher compared to the non-modified PCL scaffold (Figure 5). 

***Gene expression***

**Figure 6:**
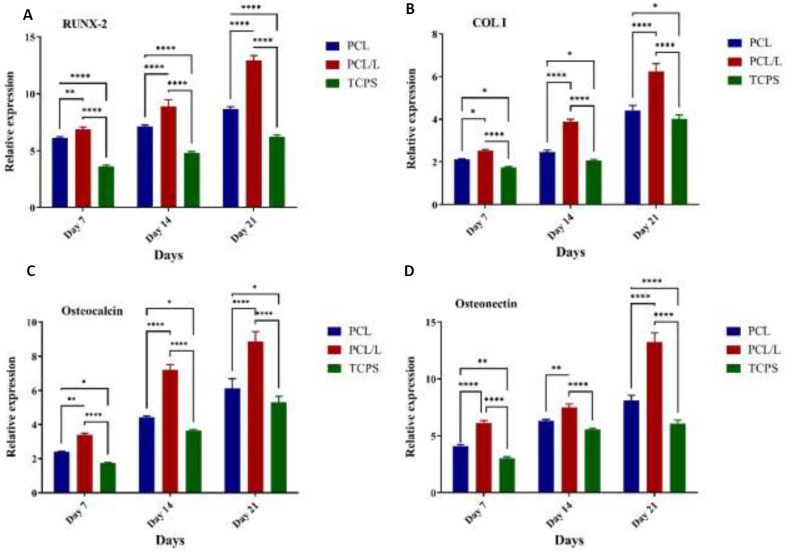
Relative expression of osteogenic genes in different time frame and treatment conditions

Real-time PCR results showed that the PCL/laminin group had a higher relative expression of the Runx2 gene than the other two groups on day 7 of induction, and this expression increased significantly on days 14 and 21. In addition, the level of collagen expression in the PCL/laminin group was higher than in the PCL and control groups on days 14 and 21. Also, similar patterns were observed in the expression of Osteocalcin and Osteonectin as the relative expression was higher in the PCL/laminin group compared to the PCL group and control group on days 7, 14, and 21 of induction (Figures 6).

## Discussion

Treating bone injuries is a major challenge in medicine. Bone tissue engineering is developed and aimed to treat bone defects such as bone fractures caused by trauma, genetic abnormalities, osteoarthritis, bone tumors, rickets, and many other diseases (18). Serious limitations such as donor complications, high surgical costs, frequent general or spinal anesthesia, and painful surgical surgery for the patient are some of the problems with surgical bone treatment or bone grafting. Therefore, the application of bone tissue engineering can be an alternative to the current surgical treatments (1, 19). 

In the present study, mesenchymal stem cells were used for bone regeneration due to their high proliferative and differentiation capacities. Consistent with our study, Abazari and colleagues showed that mesenchymal stem cells could be a major useful factor in bone tissue engineering (5). In this study, adipose tissue mesenchymal stem cells (ADMSCs) were used because of their greater availability and molecular similarity to bone mesenchymal stem cells (BMSCs) (6).

Biological implants are essential for bone regeneration. In bone tissue engineering, nanofibrous polymers act as an implant to support stem cell attachment, proliferation, differentiation, and other biological behaviors (10). It has been shown that the PCL nanofiber scaffold has excellent mechanical strength, biocompatibility, and biodegradability (6 weeks immersed in an aqueous medium) (20, 21). It has a suitable pore size and mesenchymal stem cells can be well attached to the scaffold surface. However, one of the problems with using this polymer is its low hydrophobicity, which reduces the adhesion of stem cells (21-23). 

To solve this problem, and based on a study conducted by Hojun Jeon *et al*., it is possible to coat the PCL polymer scaffolds with extracellular matrix (ECM) biomolecules. These biomolecules increase the hydrophilicity of the scaffold surface and thereby facilitate the attachment, adhesion, and differentiation of seeded cells (24). One of these biomolecules is laminin. Laminin 5 (Ln-5) plays a crucial role in the differentiation of osteogenic cells and also plays an important role in bone function (25). Laminin is composed of different components, two of which are important in bone formation, DMP-1 and SIBLING, which are involved in the differentiation of chondrocytes and osteoblasts. Research demonstrated that Ln-5 promoted the expression of Osterix, a specific bone gene. Since this gene does not express in mesenchymal stem cells in the normal state, the presence of Ln-5 induces the osteogenic differentiation of stem cells. On the other hand, Ln-5 causes cartilage to calcify, thus suppressing chondrogenesis and directing MSCs to differentiate towards osteoblasts (26). Our results showed that the surface modification of the PCL scaffold with laminin improves the physical and mechanical properties. Therefore, the presence of laminin on the PCL scaffold increases the hydrophilic properties and facilitates cell attachment to the scaffold surface through integrin receptors. In addition, due to the role of laminin in osteogenesis, the PCL/laminin scaffold improved the differentiation of ADMSCs into BMSCs (7, 27). 

The results of the MTT assay showed that the PCL/laminin scaffold had no toxicity on seeded cells and also promoted the differentiation of ADMSCs into BMSCs. Alizarin Red and Oil Red staining also indicate that ADMSCs seeded on a laminin-treated PCL scaffold have differentiated into BMSCs. Images from the inverted microscope showed that the differentiated cells are reddish-brown which was due to the calcium deposition in these cells. The results of ALP activity and calcium content assay also show that the presence of laminin on the PCL scaffold improves the efficiency and function of the scaffold in osteogenic differentiation over time. 

Also, the real-time PCR results showed the higher expression of the main osteogenic-related genes including Osteocalcin, Osteonectin, Runx2, and Collagen (Col-1) in cells differentiated on PCL/laminin (28). Therefore, the modification of the PCL scaffold with laminin has played an important role in the differentiation of ADMSCs into osteogenic stem cells. 

**Table 1:** Primer sequences used for quantitative investigation of the osteogenesis genes expression by qPCR

**Gene**	**F/R**	**Sequence**
**β2m**	Forward	TGG AAA GAA GAT ACC AAA TAT CGA
	Reverse	GAT GAT TCA GAG CTC CAT AGA GCT
**Collagen I**	Forward	TGG AGC AAG AGG CGA GAG
	Reverse	CAC CAG CAT CAC CCT TAG C
**Runx2**	Forward	GCC TTC AAG GTG GTA GCC C
	Reverse	CGT TAC CCG CCA TGA CAG TA
**Osteonectin**	Forward	AGG TAT CTG TGG GAG CTA ATC
	Reverse	ATT GCT GCA CAC CTT CTC
**Osteocalcin**	Forward	GCA AAG GTG CAG CCT TTG TG
	Reverse	GGC TCC CAG CCA TTG ATA CAG

RT-PCR, reverse transcription polymerase chain reaction

**Table 2:** PCR program for quantitative investigation of the expression of genes effective in osteogenesis

Step	Temp. °C	Time	Cycles
**Denaturation**	95	3 min	1
**Denaturation**	95	20s	40
**Annealing**	60	20s	
**Elongation**	72	20s	

Alizarin red staining showed mineral deposits in isolated cells after 20 days of differentiation (40X)

* indicates the significance between TCPS and PCL group.

*P*<0.05, TCPS: Tissue culture polystyrene; PCL: Polycaprolactone

PCL and PCL/laminin groups had higher calcium content after 21 days of differentiation compared to the 2D culture group

* indicates the significance between PCL/Laminin and TCPS also the significance between PCL and TCPS. (*P*<0.05, TCPS; tissue culture polystyrene).

TCPS: Tissue culture polystyrene; PCL: Polycaprolactone

Higher ALP activity was observed in the PCL/Laminin group after 7, 14, and 21 days of differentiation

* (*P*<0.05) & **** (*P*<0.0001) indicates the significance between PCL/Laminin and PCL or TCPS.

TCPS: Tissue culture polystyrene; ALP: Alkaline phosphatase; PCL: Polycaprolactone

Higher expression of Runx-2 (a), Col-I (b), Osteocalcin (c), and Osteonectin (d) in the PCL/laminin group was observed after 7, 14, and 21 days of differentiation. *(*P*<0.05) & **(*P*<0.01) & ****(*P*<0.0001) indicate the significance between PCL/Laminin and TCPS or PCL

TCPS: Tissue culture polystyrene; PCL: Polycaprolactone

## Conclusion

This study aimed to enhance the osteogenic differentiation of ADMSCs on a laminin-coated PCL scaffold. To improve hydrophilic properties, tensile strength, cell adhesion to the scaffold, and osteogenic differentiation, the surface of the scaffold was modified using laminin. The results of this study showed better differentiation of stem cells on the modified scaffold. It seems that laminin enhances the physicochemical properties and biocompatibility of PCL nanofiber scaffolds; and by modifying the surface of the scaffold, improves the differentiation of ADMSCs into osteogenic cells. These results paved the way for the use of laminin-modified scaffolds for use in animal and human studies in the field of bone tissue engineering.

## Authors’ Contributions

D Z, E K, and E S were involved in the search strategy and drafting. All authors supervised in all steps of the project, and revised and edited the manuscript. All authors read and approved the final manuscript.

## Compliance with Ethical Standards

Funding: This study was funded by Islamic Azad University-Mashhad Branch, Iran (1112909430902531398152895).

## Ethical approval

This article does not contain any studies with human participants or animals performed by any of the authors.

## Conflicts of Interest

The authors declare that they have no competing interests.
